# Mechanism of resveratrol-induced relaxation in the human gallbladder

**DOI:** 10.1186/s12906-017-1752-x

**Published:** 2017-05-08

**Authors:** Ching-Chung Tsai, Ming-Che Lee, Shu-Leei Tey, Ching-Wen Liu, Shih-Che Huang

**Affiliations:** 1 Department of Pediatrics, E-Da Hospital, I-Shou University, 1, Yi-Da Road, Yan-Chao, Kaohsiung City, Taiwan, Republic of China; 2 School of Chinese Medicine for Post Baccalaureate, I-Shou University, 8, Yi-Da Road, Yan-Chao, Kaohsiung City, Taiwan, Republic of China; 3 Department of General Surgery, Tzu Chi General Hospital and Tzu Chi University, 707, Section 3, Chung-Yang Road, Hualien, Taiwan, Republic of China; 4 School of Pharmacy, Kaohsiung Medical University, 100, Shih-Chuan 1st Road, Sanmin District, Kaohsiung City, Taiwan, Republic of China; 5 Department of Internal Medicine, Shosanbetsu Village Clinic, 122-8 Shosanbetsu, Shosanbetsu Village, Tomamae-Gun, Hokkaido 078-4421 Japan

**Keywords:** Resveratrol, Relaxation, Gallbladder, Human

## Abstract

**Background:**

Resveratrol is a polyphenolic compound extracted from plants and is also a constituent of red wine. Resveratrol produces relaxation of vascular smooth muscle and may prevent cardiovascular diseases. Although resveratrol has been reported to cause relaxation of the guinea pig gallbladder, limited data are available about the effect of resveratrol on the gallbladder smooth muscle in humans. The purpose of this study was to investigate the relaxation effects of resveratrol in human gallbladder muscle strips.

**Methods:**

We studied the relaxant effects of resveratrol in human gallbladder. In addition, we also investigated mechanism of resveratrol-induced relaxation in human gallbladder by tetraethylammonium (a non-selective potassium channels blocker), iberiotoxin (an inhibitor of large conductance calcium-activated potassium channel), glibenclamide (an ATP-sensitive potassium channel blocker), charybdotoxin (an inhibitor of large conductance calcium-activated potassium channels and slowly inactivating voltage-gated potassium channels), apamine (a selective inhibitor of the small conductance calcium-activated potassium channel), KT 5720 (a cAMP-dependent protein kinase A inhibitor), KT 5823 (a cGMP-dependent protein kinase G inhibitor), NG-Nitro-L-arginine (a competitive inhibitor of nitric oxide synthase), tetrodotoxin (a selective neuronal Na^+^ channel blocker), and ω-conotoxin GVIA (a selective neuronal Ca^2+^ channel blocker).

**Results:**

The present study showed that resveratrol has relaxant effects in human gallbladder muscle strips. In addition, we found that resveratrol-induced relaxation in human gallbladder is associated with nitric oxide, ATP-sensitive potassium channel, and large conductance calcium-activated potassium channel pathways.

**Conclusions:**

This study provides the first evidence concerning the relaxant effects of resveratrol in human gallbladder muscle strips. Furthermore, these results demonstrate that resveratrol is a potential new drug or health supplement in the treatment of biliary colic.

## Background

Resveratrol (3′,5′,4′-trihydroxystilbene) is a naturally occurring polyphenolic compound that was first isolated from the roots of white Hellebore [1]. It has been found in various plant roots and also a constituent of red wine [2, 3]. Studies have shown that resveratrol may have a number of health benefits. Resveratrol has shown potential beneficial effects in cardiovascular disease in preclinical studies. The vasorelaxant activity of resveratrol occurs via activation of adenosine monophosphate activated protein kinase (AMPK), nuclear factor erythroid-2 related factor 2 (NRF2), and silent information regulator 1 (SIRT1) [4, 5]. In addition, resveratrol has antioxidant, anti-inflammatory, and cytoprotective effects [6].

Biliary colic is most frequently associated with obstruction caused by gallstone impaction in the cystic duct or the common bile duct. If the biliary duct obstruction persists, the gallbladder becomes inflamed. Medical treatment of biliary colic includes nonsteroidal anti-inflammatory drugs (NSAIDs) such as ketorolac and diclofenac, as well as anti-cholinergic drugs such as scopolamine or propantheline [7, 8]. However, NSAIDs usually induce peptic ulcers or gastrointestinal upset, and common side effects of anti-cholinergic drugs include urinary retention, glaucoma, and confusion [9].

Although resveratrol produces relaxation of the guinea pig gallbladder muscle, there is limited data concerning the effect of resveratrol on the gallbladder smooth muscle in humans [10, 11]. The objective of this study was to investigate the effect of resveratrol in human gallbladder smooth muscle and the underlying mechanism.

## Methods

Tetraethylammonium (TEA) was purchased from Santa Cruz Biotech, California, USA. Iberiotoxin was purchased from Alomone, Jerusalem, Israel. Glibenclamide was purchased from Research Biochemical International, Massachusetts, USA. Tetrodotoxin (TTX) was purchased from Tocris Bioscience, Bristol, UK. ω-conotoxin GVIA (CTX) was purchased from Bachem, Bubendorf, Switzerland. KT 5720, KT 5823, NG-Nitro-L-arginine (L-NNA), apamine, papaverine, and resveratrol were obtained from Sigma-Aldrich, Missouri, USA. Charybdotoxin was purchased from AnaSpec Inc., California, USA.

### The effects of resveratrol in human gallbladder

All procedures were performed in accordance with relevant laws and institutional guidelines. In addition, the Institutional Review Board / Ethics Committee of Tzu Chi Hospital approved the protocol for this work (approval number: IRB097-55). The human specimens of gallbladder were acquired from patients undergoing cholecystectomy for gallstone (excluding acute cholecystitis) or hepatoma, and informed consent was obtained from every patient. Human gallbladder specimens were acquired from 39 patients (The ratio of male to female was 22:17; the age range was from 28 to 80 years; the median age was 58 years; the mean age was 56.8 ± 1.9 years). A standard incubation solution was made up of the following composition (in mM): 118 NaCl, 25 NaHCO_3_, 4.7 KCl, 14 glucose, 1.2 NaH_2_PO_4_, 1.8 CaCl_2_, pH 7.4. Immediately after surgical removal of the gallbladder, an area of 3 × 5 cm was excised from the middle portion of each gallbladder corpus. The human specimens were placed in oxygenated standard incubation solution gassed with 95% O_2_ + 5% CO_2_ for transportation to the laboratory, where the relaxation experiment was at once initiated. The period of transportation was less than 30 min. The human gallbladder strips were used to study the effects of resveratrol on gallbladder. The muscle strips, which were 3 mm wide and 10 mm long, were trimmed and hanged in 7 ml organ baths containing a standard incubation solution, incubated at 37 °C, and continuously gassed with 95% O_2_ + 5% CO_2_. Subsequently, the muscle strips were joined to isometric force transducers (FT.03; Grass Technologies, West Warwick, RI, USA), which were linked to amplifiers and a computer recording system (BIOPAC Systems, CA, USA). The basal tension of the muscle strips was set at 1.0 g. After a 30 min equilibration period, carbachol (1 μM) was added into the organ bath, the muscle strip contractions were measured, and the carbachol was washed out. For measurements of relaxation in carbachol-precontracted strips, resveratrol was added to muscle strips 15 min after the addition of carbachol. Resveratrol was added in a noncumulative fashion, i.e., with single dose administration (1 μM, 10 μM, 30 μM, 100 μM, or 1 mM, n is at least ≥4), and the isolated gallbladder muscle strip relaxations were measured. After the final step of the experiments, papaverine (100 μM) was added, and the muscle strip relaxations were measured. The difference between papaverine-induced relaxation and carbachol-induced contraction served as a reference (100%) for the relaxation response to resveratrol.

### The effects of resveratrol on neurally-mediated human gallbladder relaxation

In order to determine whether resveratrol triggers neurally-mediated human gallbladder relaxation, 1 μM TTX (a neuronal Na^+^ channel blocker) and 1 μM CTX (a neuronal Ca^2+^ channel blocker) were added 15 min before the addition of 100 μM resveratrol to test the effects of resveratrol on neurally-mediated human gallbladder relaxation.

### The effects of cAMP, cGMP and nitric oxide (NO) on resveratrol-induced human gallbladder relaxation

In order to investigate the mechanism of resveratrol-induced human gallbladder relaxation, 1 μM KT 5720 (a cyclic adenosine monophosphate (cAMP)-dependent protein kinase (PKA) inhibitor), 1 μM KT 5823 (a cyclic guanosine monophosphate (cGMP)-dependent protein kinase (PKG) inhibitor), and 100 μM L-NNA (a competitive nitric oxide synthase (NOS) inhibitor) were added 30 min before the addition of 100 μM resveratrol to test the involvement of cAMP, cGMP, and NO on resveratrol-induced human gallbladder relaxation [12, 13].

### The effects of potassium channel antagonists on resveratrol-induced human gallbladder relaxation

In order to investigate whether potassium channels are involved in resveratrol-induced human gallbladder relaxation, 1 mM TEA (a non-selective potassium channel blocker), 200 nM iberiotoxin (an inhibitor of large conductance calcium-activated potassium (BKCa) channels), 100 nM charybdotoxin, 200 nM apamine, and 10 μM glibenclamide (an adenosine triphosphate (ATP)-sensitive potassium channel blocker) were added 30 min before the addition of 100 μM resveratrol to test the effects of potassium channels on resveratrol-induced human gallbladder relaxation [14–17].

### Data analysis

Results are expressed as the means ± SEMs. Sample size is at least ≥3 in each subgroup. Statistical evaluation was applied using Student’s t-test or one-way analysis of variance (ANOVA), followed by Dunnett’s test. *P* < 0.05 was considered statistically significant.

## Results

### The effects of resveratrol in human gallbladder

**Fig. 1:**
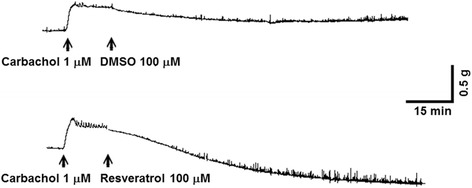
Typical relaxant tracings of human gallbladder muscle strips induced by resveratrol. The arrows indicate the addition of carbachol, resveratrol and dimethyl sulfoxide (DMSO)

**Fig. 2:**
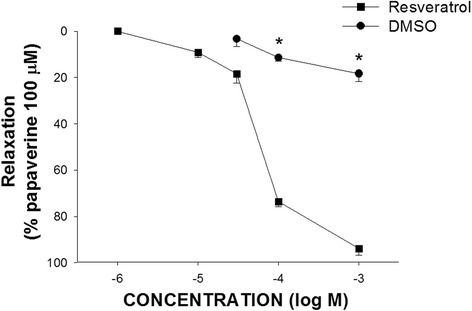
The ability of resveratrol or dimethyl sulfoxide (vehicle) to induce relaxation of human gallbladder muscle strips. Resveratrol-induced relaxation in the human gallbladder muscle fiber in a dose-dependent manner. The values are expressed as a percent of a papaverine (100 μM)-induced relaxation. The results given are from at least four experiments. The vertical bars represent ± standard error of the mean (SEM). * represents a significant difference compared with the relaxation induced by resveratrol (*p* < 0.05)

In muscle strips isolated from human gallbladder specimens, 1 μM carbachol induced a marked and long-duration muscle contraction and 1 mM resveratrol induced an obvious muscle relaxation of the carbachol precontracted gallbladder strips (Fig. 1). The resveratrol-induced relaxation in human gallbladder was dose-dependent (Fig. 2). Resveratrol caused detectable relaxation of human gallbladder muscle strips at 10 μM and maximal relaxation at 1 mM. The maximal relaxation caused by 1 mM resveratrol was 90.8 ± 5.1% (*n* = 6) of the relaxation caused by 100 μM papaverine.

### The effects of resveratrol on neurally-mediated human gallbladder relaxation

**Fig. 3:**
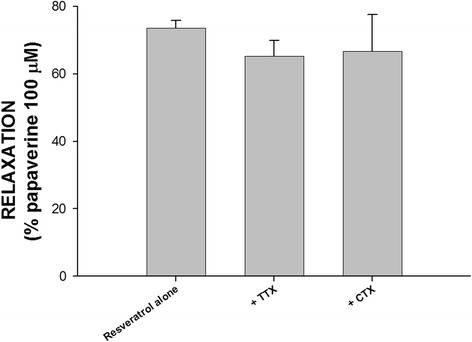
The effects of tetrodotoxin (TTX) and ω-conotoxin GVIA (CTX) on resveratrol-induced relaxation in the human gallbladder. TTX (1 μM) and CTX (1 μM) had no significant effect on resveratrol-induced relaxation of human gallbladder muscle strips. The results shown are from at least three experiments. The vertical bars represent ± standard error of the mean (SEM)

As shown in Fig. 3, neither 1 mM TTX nor 1 mM CTX had inhibitory effects on the relaxation in human gallbladder induced by resveratrol (*p* > 0.05, *n* = 6 and 3, respectively). These results indicate that resveratrol-induced human gallbladder relaxation does not involve the activation of the enteric nervous system.

### The effects of cAMP, cGMP and NO on resveratrol-induced human gallbladder relaxation

**Fig. 4:**
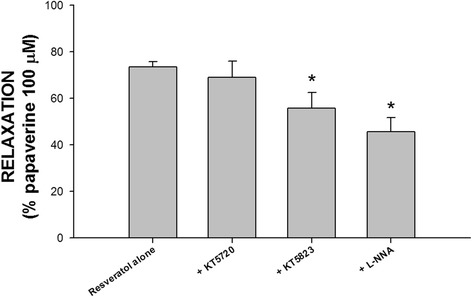
The effects of KT 5720, KT 5823, and NG-nitro-L-arginine (L-NNA) on resveratrol-induced relaxation in the human gallbladder. KT 5720 (1 μM) had no significant effect on resveratrol-induced relaxation of human gallbladder muscle strips (*p* > 0.05, *n* = 6). In contrast, L-NNA (100 μM) and KT 5823 (1 μM) exerted significant inhibitory effects on resveratrol-induced relaxation of human gallbladder muscle strips (*p* < 0.05, *n* = 6). The vertical bars represent ± standard error of the mean (SEM)

As shown in Fig. 4, 1 μM KT 5720 (*n* = 6) had no inhibitory effects on the relaxation in human gallbladder induced by 100 μM of resveratrol (*p* > 0.05). However, both 1 μM KT 5823 and 100 μM L-NNA had significant inhibitory effects on resveratrol-induced human gallbladder relaxation (*p* < 0.05, *n* = 5 and 6 respectively). These results indicate that the relaxation in human gallbladder induced by resveratrol is associated with cGMP and NO production.

### The effects of potassium channel antagonists on resveratrol-induced human gallbladder relaxation

**Fig. 5:**
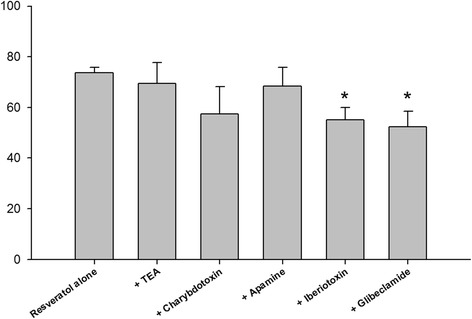
The effects of tetraethylammonium (TEA), charybdotoxin, apamine, iberiotoxin, and glibenclamide on resveratrol-induced relaxation in the human gallbladder. TEA (1 mM), apamine (100 nM), and charybdotoxin (100 nM) had no significant effect on resveratrol-induced relaxation of human gallbladder muscle strips (*p* > 0.05, *n* ≥ 4). In contrast, iberiotoxin (200 nM) and glibenclamide (10 μM) significantly inhibited resveratrol-induced relaxation of human gallbladder muscle strips (*p* < 0.05, *n* = 7). The vertical bars represent ± standard error of the mean (SEM)

As shown in Fig. 5, 1 mM TEA (*n* = 5) and 200 nM apamine (*n* = 4) did not inhibit the relaxation in human gallbladder induced by 10 μM of resveratrol (*p* > 0.05, *n* = 12 in the resveratrol group). However, 100 nM charybdotoxin (*n* = 5) had a trend to inhibit relaxation in human gallbladder induced by 10 μM of resveratrol (*p* = 0.093). Furthermore, 10 μM of glibenclamide (*n* = 6) and 200 nM iberiotoxin (*n* = 6) induced a significant inhibitory effect on resveratrol-induced human gallbladder relaxation (*p* < 0.05). These results indicate that the relaxant effects in human gallbladder induced by resveratrol are related to ATP-sensitive potassium channels and BKCa channels.

## Discussion

The relaxation process of smooth muscle is associated with a decreased concentration of intracellular calcium and increased activity of myosin light chain phosphatase. In addition, the relaxation of smooth muscle is regulated by many signaling pathways, including NO, cAMP, cGMP, and K^+^ channels [18].

Previous studies have shown that resveratrol-induced vasorelaxant activity was mainly associated with NO and potassium channels [19–21]. In this study, we showed that resveratrol can induce human gallbladder muscle fiber relaxation in a dose-dependent manner and we also investigated the mechanisms of resveratrol-induced relaxation in human gallbladder.

TTX is a selective blocker of neuronal Na^+^ channels, and CTX is a blocker of neuronal Ca^2+^ channels. In order to determine whether resveratrol triggers neurally-mediated human gallbladder relaxation, the effects of TTX and CTX on resveratrol-induced relaxation of the gallbladder were examined in this study. TTX and CTX did not inhibit the resveratrol-induced relaxation in human gallbladder. These results suggested that resveratrol-induced relaxation in human gallbladder is not involved in neurally-mediated human gallbladder relaxation.

In addition, L-NNA is a competitive NOS inhibitor and can suppress neuronal NOS (nNOS) and endothelial NOS (eNOS) [22, 23]. L-NNA was applied to investigate whether resveratrol affects the nNOS of the neuron or eNOS expressed in smooth muscle cells, ultimately leading to relaxation. Resveratrol-induced relaxation in human gallbladder was inhibited by L-NNA, indicating that resveratrol-induced relaxation in human gallbladder is mediated either by the nNOS in nitrergic nerves in the gallbladder or eNOS expressed in smooth muscle cells or both [24–26]. KT 5823 is an inhibitor of PKG, and KT 5720 is an inhibitor of PKA. KT 5823 inhibited resveratrol-induced relaxation in human gallbladder. However, KT 5720 was unable to inhibit resveratrol-induced relaxation in human gallbladder. These results suggested that resveratrol-induced relaxation in human gallbladder is related to PKG, but not PKA pathway.

The activation of membrane K^+^ channels leads to hyperpolarization of the plasma membrane and further suppresses Ca^2+^ influx into the cell, giving rise to relaxation of smooth muscle [27]. Charybdotoxin is an inhibitor of BKCa channels and slowly inactivating voltage-gated potassium channels, iberiotoxin is an inhibitor of BKCa channels, apamine is a selective inhibitor of small conductance calcium-activated potassium channels, TEA is a non-selective inhibitor of potassium channel, and glibenclamide is an inhibitor of ATP-sensitive potassium channel [14–17]. Our results show that both glibenclamide and iberiotoxin can significantly inhibit resveratrol-induced relaxation in human gallbladder, indicating that resveratrol affects ATP-sensitive potassium channels and BKCa channels to facilitate resveratrol-induced relaxant pathways in human gallbladder. In addition, ATP-sensitive potassium channels are connected with the resveratrol-induced relaxation of the human umbilical vein and the rat corpus cavernosum [28, 29].

Resveratrol-induced relaxation of cholecystokinin octapeptide- or KCl-induced tension in guinea pig gallbladder strips mainly occurs via the L-type Ca^2+^ channels [10]. The difference of resveratrol-induced relaxation mechanisms between the guinea pig and human gallbladders might be affected by different contractile stimulants and species, and more studies are needed to clarify the difference between them.

## Conclusions

In conclusion, the present study showed that resveratrol-induced relaxation of gallbladder occurs through NO, ATP-sensitive potassium channel, and large conductance calcium-activated potassium channel pathways. These results suggest that resveratrol is a potential new drug in the treatment of biliary colic or other gastrointestinal colicky pain.

## Abbreviations

AMPK: Adenosine monophosphate activated protein kinase
ANOVA: Analysis of variance
ATP: Adenosine triphosphate
BKCa: Large conductance calcium-activated potassium channels
cAMP: Cyclic adenosine monophosphate
cGMP: Cyclic guanosine monophosphate
CTX: ω-conotoxin GVIA
eNOS: endothelial NOS
L-NNA: NG-Nitro-L-arginine
nNOS: neuronal NOS
NO: Nitric oxide
NRF2: Nuclear factor erythroid-2 related factor 2
NSAIDs: Nonsteroidal anti-inflammatory drugs
PKA: Cyclic adenosine monophosphate-dependent protein kinase
PKG: Cyclic guanosine monophosphate-dependent protein kinase
SIRT1: Silent information regulator 1
TEA: Tetraethylammonium
TTX: Tetrodotoxin

## References

[1] Catalgol B, Batirel S, Taga Y, Ozer NK (2012). Resveratrol: French paradox revisited. Front Pharmacol.

[2] Mattivi FZ (1993). Solid phase extraction of trans-resveratrol from wines for HPLC analysis. Lebensm Unters Forsch.

[3] Guerrero RF, García-Parrilla MC, Puertas B, Cantos-Villar E (2009). Wine, resveratrol and health: a review. Nat Prod Commun.

[4] Zordoky BN, Robertson IM, Dyck JR. Preclinical and clinical evidence for the role of resveratrol in the treatment of cardiovascular diseases. Biochim Biophys Acta. 2015;1852:1155–77. 10.1016/j.bbadis.2014.10.016 25451966

[5] Bonnefont-Rousselot D (2016). Resveratrol and Cardiovascular Diseases. Nutrients.

[6] Diaz-Gerevini GT, Repossi G, Dain A, Tarres MC, Das UN, Eynard AR (2016). Beneficial action of resveratrol: how and why?. Nutrition.

[7] Colli A, Conte D, Valle SD, Sciola V, Fraquelli M (2012). Meta-analysis: nonsteroidal anti-inflammatory drugs in biliary colic. Aliment Pharmacol Ther.

[8] Henderson SO, Swadron S, Newton E (2002). Comparison of intravenous ketorolac and meperidine in the treatment of biliary colic. J Emerg Med.

[9] Ness J, Hoth A, Barnett MJ, Shorr RI, Kaboli PJ (2006). Anticholinergic medications in community-dwelling older veterans: prevalence of anticholinergic symptoms, symptom burden, and adverse drug events. Am J Geriatr Pharmacother.

[10] Kline LW, Karpinski E. The resveratrol-induced relaxation of cholecystokinin octapeptide- or KCl-induced tension in male guinea pig gallbladder strips is mediated through L-type Ca^2+^ channels. J Neurogastroenterol Motil. 2015;21:62–8. 10.5056/jnm14093 PMC4288087 25537678

[11] Wang LD, Qiu XQ, Tian ZF, Zhang YF, Li HF (2008). Inhibitory effects of genistein and resveratrol on guinea pig gallbladder contractility *in vitro*. World J Gastroenterol.

[12] Jing F, Liu M, Yang N, Liu Y, Li X, Li JJ (2013). Relaxant effect of chloroquine in rat ileum: possible involvement of nitric oxide and BKCa. Pharm Pharmacol.

[13] Huang SC. Endothelin A receptors mediate relaxation of guinea pig internal anal sphincter through cGMP pathway. Neurogastroenterol Motil. 2010;22:1009–16. 10.1111/j.1365-2982.2010.01513.x 20465591

[14] Giangiacomo KM, Sugg EE, Garcia-Calvo M, Leonard RJ, McManus OB, Kaczorowski GJ, Garcia ML (1993). Synthetic charybdotoxin-iberiotoxin chimeric peptides define toxin binding sites on calcium-activated and voltage-dependent potassium channels. Biochemistry.

[15] Wittekindt OH, Visan V, Tomita H, Imtiaz F, Gargus JJ, Lehmann-Horn F, Grissmer S, Morris-Rosendahl DJ (2004). An apamin- and scyllatoxin-insensitive isoform of the human SK3 channel. Mol Pharmacol.

[16] Weatherall KL, Goodchild SJ, Jane DE, Marrion NV (2010). Small conductance calcium-activated potassium channels: from structure to function. Prog Neurobiol.

[17] Zhang J, Halm ST, Halm DR (2012). Role of the BK channel (KCa1.1) during activation of electrogenic K^+^ secretion in guinea pig distal colon. Am J Physiol Gastrointest Liver Physiol.

[18] Matsuda NM, Miller SM (2010). Non-adrenergic non-cholinergic inhibition of gastrointestinal smooth muscle and its intracellular mechanism(s). Fundam Clin Pharmacol.

[19] Novakovic A, Bukarica LG, Kanjuh V, Heinle H (2006). Potassium channels-mediated vasorelaxation of rat aorta induced by resveratrol. Basic Clin Pharmacol Toxicol.

[20] Nagaoka T, Hein TW, Yoshida A, Kuo L (2007). Resveratrol, a component of red wine, elicits dilation of isolated porcine retinal arterioles: role of nitric oxide and potassium channels. Invest Ophthalmol Vis Sci.

[21] Pullen C, Coulson FR, Fenning A (2014). Effects of resveratrol and nebivolol on isolated vascular and cardiac tissues from young rats. Adv Pharmacol Sci.

[22] Furfine ES, Harmon MF, Paith JE, Garvey EP (1993). Selective inhibition of constitutive nitric oxide synthase by L-NG-nitroarginine. Biochemistry.

[23] Garvey EP, Tuttle JV, Covington K, Merrill BM, Wood ER, Baylis SA, Charles IG (1994). Purification and characterization of the constitutive nitric oxide synthase from human placenta. Arch Biochem Biophys.

[24] Murthy KS, Teng B, Jin J, Makhlouf GM (1998). G protein-dependent activation of smooth muscle eNOS via natriuretic peptide clearance receptor. Am J Phys.

[25] Teng B, Murthy KS, Kuemmerle JF, Grider JR, Sase K, Michel T, Makhlouf GM (1998). Expression of endothelial nitric oxide synthase in human and rabbit gastrointestinal smooth muscle cells. Am J Phys.

[26] Makhlouf GM, Murthy KS, Podolsky DK, Camilleri M, Fitz JG, Kalloo AN, Shanahan F, Wang TC (2016). Smooth muscle of the gut. Yamada's textbook of gastroenterology.

[27] Hill-Eubanks DC, Werner ME, Heppner TJ, Nelson MT (2011). Calcium signaling in smooth muscle. Cold Spring Harb Perspect Biol.

[28] Protić D, Beleslin-Čokić B, Spremović-Rađenović S, Radunović N, Heinle H, Sćepanović R, Gojković Bukarica L (2014). The different effects of resveratrol and naringenin on isolated human umbilical vein: the role of ATP-sensitive K^+^ channels. Phytother Res.

[29] Dalaklioglu S, Ozbey G (2014). Role of different types of potassium channels in the relaxation of corpus cavernosum induced by resveratrol. Pharmacogn Mag.

